# Therapeutic Potential of Mesenchymal Stem Cells in Psoriasis

**DOI:** 10.1007/s12013-025-01843-x

**Published:** 2025-08-01

**Authors:** Rebecca Shin Yee Wong, Kien Hui Chua, Ee Wern Tan, Bey Hing Goh

**Affiliations:** 1 Department of Medical Education, Sir Jeffrey Cheah Sunway Medical School, Faculty of Medical and Life Sciences, Sunway University, 5, Jalan Universiti, Bandar Sunway, 47500 Petaling Jaya, Selangor Malaysia; 2 Department of Physiology, Faculty of Medicine, Universiti Kebangsaan Malaysia, Jalan Yaacob Latif, Bandar Tun Razak, 56000 Cheras, Kuala Lumpur Malaysia; 3 Sunway Biofunctional Molecules Discovery Centre, Faculty of Medical and Life Sciences, Sunway University, 5, Jalan Universiti, Bandar Sunway, 47500 Petaling Jaya, Selangor Malaysia; 4 Faculty of Health, Australian Research Centre in Complementary and Integrative Medicine, University of Technology Sydney, Ultimo, Australia; 5 Graduate Institute of Cancer Biology and Drug Discovery, College of Medical Science and Technology, Taipei Medical University, Taipei, Taiwan

**Keywords:** Mesenchymal stem cells, Immunomodulation, Secretome, Psoriasis, Cell-based therapy

## Abstract

Psoriasis is a chronic immune-mediated disease mainly affecting the skin with different clinical manifestations. As patients with psoriasis may also suffer from psoriatic arthritis and the skin lesions of psoriasis are disfiguring, their quality of life is often impaired. Many environmental and genetic factors have been implicated in psoriasis development. Currently, there is no cure for the disease and long-term drug treatment is usually necessary, especially in moderate to severe cases. Mesenchymal stem cells (MSCs) are popular candidates for cell-based treatment in many immune-mediated diseases due to their ability to secrete a wide array of cytokines and growth factors and their immunomodulatory properties. MSCs from various sources administered via different routes have been shown to ameliorate psoriasis. This review gives an overview of psoriasis and MSCs and examine preclinical and clinical studies concerning the application of MSCs in the treatment of psoriasis, as well as consolidate major findings in this area of research.

## Introduction

Psoriasis is a chronic inflammatory disease affecting the skin whereas about 20–30% of patients who have psoriasis also have psoriatic arthritis [1, 2]. Psoriasis is generally considered a genetic disease and its pathogenesis is multifactorial, commonly triggered by environmental factors. Several clinical types of psoriasis exist and they can be broadly classified into non-pustular and pustular psoriasis. The classic plaque psoriasis (also called psoriasis vulgaris) comprises about 90% of all psoriasis cases and is characterised by well-demarcated, dry, itchy symmetric and scaly erythematous plaques [3, 4].

Owing to the chronic and relapsing nature of the condition, long-term treatment is often necessary for patients with psoriasis. However, to this end, there is no cure for these conditions and pharmacotherapy remains the mainstay of treatment. Mild to moderate disease can be treated with steroids, vitamin D, and phototherapy while severe disease requires systemic treatment such as methotrexate, cyclosporin, or biologics (e.g. infliximab, etanercept). Non-steroidal inflammatory drugs (NSAIDs), on the other hand, are used to reduce inflammation and relieve pain in psoriatic arthritis [5].

Mesenchymal stem cells (MSCs) are a type of stem cell with multipotent differentiation capacity that reside in many locations in the body such as adipose tissue, bone marrow, dental pulp, and umbilical cord [6]. MSCs are very popular among researchers and clinicians because they can be readily sourced from the body, grown in the laboratory, as well as differentiated into various cell types [7]. Besides differentiating into cells that originate from the mesoderm (e.g. osteoblasts, adipocytes, and chondrocytes) [8], earlier research showed that MSCs also possess the capacity to transdifferentiate into cells that exhibit endodermal and neuroectodermal properties [9].

The immunomodulatory and immunosuppressive effects exerted by MSCs and the wide array of secretory products (e.g. cytokines and growth factors) produced by MSCs have added to their popularity, making MSCs promising treatment options for immune-related diseases. Several studies have reported the use of MSCs in immune-mediated diseases including Crohn’s disease, rheumatoid arthritis (RA), multiple sclerosis (MS), systemic lupus erythematosus (SLE), type 1 diabetes mellitus etc [10]. There are numerous reviews on the use of MSCs in common autoimmune diseases like RA and SLE in the published literature. Comparatively, fewer reviews have focused on psoriasis. This review will give an overview of psoriasis and MSCs and consolidate key findings of recent in vitro, animal, and clinical studies that used MSCs in the treatment of psoriasis.

## Overview of Psoriasis

The World Health Organization (WHO) considers psoriasis a serious and disfiguring disease that imposes a significant challenge on public health owing to its social, economic and psychological burden [11]. The prevalence of psoriasis varies widely, depending on the geographical regions. In a systematic analysis, Rosa et al. [12] reported the prevalence of psoriasis to be 0.14% in East Asia, 1.10% in high-income Southern Latin America, 1.50% in North America, 1.83% in Central Europe, 1.92% in Western Europe and 1.99% in Australasia. In another systematic review, two peaks in the onset of psoriasis were observed. The first peak was seen in people around 30–39 years of age while the second peak, in those around 60–69 years of age. Although there were no gender differences in the incidence and prevalence of psoriasis, some studies reported a slight male predominance, whereas the disease presents slightly earlier in females [13].

## Risk Factors for Psoriasis

This section discusses the risk factors for psoriasis as many environmental and genetic factors play a crucial role in the development, trigger, and exacerbation of psoriasis. An association between some medical conditions and an increased risk of psoriasis development has also been reported in the published literature.

### Genetic Factors

Earlier genetic studies on psoriasis depended very much on linkage analysis and the identification of candidate genes. However, more recent research has applied exome-wide association study (EWAS), Immunochip study and genome-wide association study (GWAS) to identify susceptible gene loci. In a large-scale study involving 10,725 pairs of twins from the Danish Twin Registry, it was reported that monozygotic twins had a larger proband-wise concordance (0.33) for psoriasis compared to that of dizygotic twins (0.17). Sixty-eight percent of the observed variations in psoriasis susceptibility was due to genetic factors, while the remaining variations were due to non-shared environmental factors. Hence, the study concluded that psoriasis is a complex and multifactorial condition attributed to a mixture of exogenous and endogenous factors [14]. Based on the Online Mendelian Inheritance in Man (OMIM), several psoriasis susceptible (PSORS) gene loci have been identified thus far. These include PSORS1–15, of which PSORS1 has the strongest link and effect. A detailed discussion of these susceptible gene loci is beyond the scope of this review. Table 1 summarizes the psoriasis susceptible gene loci, their chromosomal location, and the genes or markers involved [15–19].

**Table 1:** Psoriasis Susceptible Gene Loci and Genes Involved

PSORS	Location	Gene/markers	Remarks
PSORS1	6p21.33	*HLA-C, CCHCR1* and *CDSN* genes	Particularly, allele *HLA-Cw6* is highly associated with psoriasis
PSORS2	17q25.3	Mutation in *CARD14* gene	Also observed in two conditions i.e. pityriasis rubra pilaris and generalized pustular psoriasis other than plaque-psoriasis
PSORS3	4q	*IRF2 gene; IL2* gene*; IL21* gene	*IL2* and *IL21* genes are associated with psoriatic arthritis *IL21* gene associated with epidermal hyperplasia in psoriasis
PSORS4	1q21	Deletion in *LCE3B* and *LCE3C* genes	Part of Epidermal Differentiation Cluster (EDC). May be related to disruption of skin barrier function
PSORS5	3q21	*SLC12A8* gene	A susceptibility locus for psoriasis vulgaris
PSORS6	19p13	*BSG* gene; *JunB* gene	T allele of BSG gene shown to have a decreased association with psoriasis while A allele shown to an increased association. *JunB* gene shown to be associated with hallmarks of psoriasis and psoriatic arthritis in animal model
PSORS7	1p	*IL12B* gene; IL23R gene	Both genes are susceptibility loci for psoriasis and psoriatic arthritis
PSORS8	16q	NOD2/CARD15 gene	Gene overlaps with susceptibility locus of Crohn’s disease. However, some studies found lack of association between CARD15 and psoriasis
PSORS9	4q31	D4S1597 marker	Susceptibility to psoriasis vulgaris in a Chinese Han population.
PSORS10	18p11	Locus between markers D18S63 and D18S967	Psoriasis susceptibility on 18p11 supported by haplotype association analysis
PSORS11	5q31-q33	*IL12B* gene; *IL23R* gene; *TNIP1* gene	All three genes have a role in inflammatory processes
PSORS12	20q13	*RNF114* gene	Regulates pathways underlying epithelial inflammation
PSORS13	6q21	*TRAF3IP2* gene	Demonstrated shared susceptibility for psoriatic arthritis and psoriasis vulgaris
PSORS14	2q14	Mutation in *IL36RN* gene	Associated with pustular psoriasis
PSORS15	2q36	*AP1S3* gene	Heterozygous mutation associated with pustular psoriasis

#### Immunological factors

Psoriasis is primarily driven by dysregulated immune responses involving both the innate and adaptive arms of the immune system. Recent studies emphasize the pivotal role of T helper Th-17 and Th-1 cells in the pathogenesis of psoriasis, with interleukin IL-17A, IL-23, and tumor necrosis factor-alpha (TNF-α) serving as central mediators of skin inflammation [20]. Keratinocytes, once thought to be passive targets, are now recognized as active participants that respond to these cytokines by producing antimicrobial peptides (AMPs), chemokines, and additional pro-inflammatory mediators, thereby perpetuating immune cell recruitment and inflammation [21]. Dendritic cells (DCs), particularly plasmacytoid DCs, initiate this cycle by producing interferon-alpha (IFN-α), which activates myeloid DCs to secrete IL-12 and IL-23, favoring Th-1 and Th-17 differentiation, respectively [22].

Furthermore, innate immune cells such as γδ T cells, innate lymphoid cells (ILCs), and neutrophils also contribute significantly to disease progression. γδ T cells are potent producers of IL-17 in psoriatic lesions, while ILC3s enhance local inflammation through IL-22 production [23]. A defective regulatory T cell (Treg) response, combined with heightened Th-17 and Th-1 activity, results in a chronic pro-inflammatory environment that sustains the disease [24]. These insights into immune dysregulation have informed the development of targeted biologic therapies and provide a rational basis for exploring MSCs, given their known ability to modulate T cell subsets, inhibit DC maturation, and restore immune tolerance.

#### Environmental factors

Several environmental or extrinsic factors have been linked to the trigger or exacerbation of psoriasis. For example, mechanical stress is related to the Koebner phenomenon, which refers to the formation of new psoriatic lesions in previously healthy skin post-injury or trauma [25]. Several agents or factors such as drugs, radiotherapy, trauma to skin, surgical incision, tattooing, exposure to sunlight etc. may trigger development of new psoriatic lesions [26]. Some drugs, such as beta-blockers, lithium, NSAIDs, tetracycline and synthetic antimalarials have apparent causal relationship with psoriasis while others like ACE inhibitors, interferons and some antifungals have been associated with psoriasis but the relationship is less well-defined [27].

Some studies have reported the association between psoriasis and vaccination. For example, COVID-19 vaccine has been linked to the flare-up of psoriasis [28]. Psoriatic skin lesions have also been reported after Bacillus Calmette–Guerin (BCG) vaccination [29], influenza vaccination [30] and tetanus-diphtheria vaccination [31]. Other triggering factors that for flare-ups of psoriasis include stress, tobacco, alcohol, infections and hormonal changes [32].

#### Other factors

Other than genetic and environmental factors, certain conditions are more prevalent in patients with psoriasis or increase the risk of psoriasis. Obesity, for instance, predisposes patients to psoriasis and amplify inflammation in psoriasis. An earlier study demonstrated that adiposity and weight gain increased the risk of psoriasis development. People who are obese, with a body mass index ≥35 showed a relative increased risk of 2.69 when compared to those who are lean [33]. Another study reported a two-fold increased risk of psoriasis in people who were obese or having a high abdominal fat mass [34]. Patients with diabetes mellitus (DM) are also more likely to have psoriasis and research has shown an increased prevalence of type 2 DM among psoriatic patients when compared to controls [35]. Psoriasis has also been associated with hypertension [36] and dyslipidemia [37].

## Clinical Features of Psoriasis

The type of psoriasis determines the clinical presentation of psoriasis. This section gives an overview of the key clinical features of various types of psoriasis, as well as co-morbidities associated with psoriasis. In general, psoriasis can be broadly divided into non-pustular or pustular psoriasis and subtypes exist for each category. Wide variations of skin manifestations are observed in psoriasis and different forms can co-exist at the same time. Table 2 summarizes the key features of each type of psoriasis [38–42].

**Table 2:** Clinical Features of Different Types of Psoriasis

Non-pustular psoriasis	
Type	Key clinical features
Psoriasis vulgaris	• Also known as plaque psoriasis • 90% of psoriasis patients have psoriasis vulgaris • Well-demarcated, dry, itchy symmetric and scaly erythematous plaques • Lesions can be painful and flaking and bleeding may occur if the scales are removed • Commonly found on the scalp, extensor surfaces of the limbs and trunk • Can be further classified based on site of lesions (e.g. flexural, palmoplantar, scalp and nail psoriasis)
Guttate psoriasis	• About 2% of psoriasis • Commonly seen in children, adolescents and young adults • Characterized by droplet-like small papules on the limbs and trunk • May be triggered by streptococcal infection and later develop life-long psoriasis vulgaris
Erythrodermic psoriasis	• An acute condition that affects a large surface of the body (usually >80%), either due to progressive development of chronic plague psoriasis or triggered by drugs, hypothermia, steroid withdrawal or infections etc. • Life-threatening and requires urgent treatment
Inverse psoriasis	• Also referred to as flexural or intertriginous psoriasis • Psoriasis that involves the skin folds or flexure • Characterized by symmetric, fissured plaques with sharp contours
Pustular psoriasis	
Localized pustular psoriasis	• Two distinct forms: ➢ Psoriasis pustulosa palmoplantaris- lesions are mainly found on the palms and soles ➢ Acrodermatitis continua of Hallopeau- lesions are found on the nails and tips of fingers and toes
Generalized pustular psoriasis	• Rare and serious form of psoriasis • Characterized by widespread sterile pustules on inflamed skin • May develop independently or secondary to infections, drugs or abrupt steroid withdrawal, hypocalcaemia etc. • Impetigo herpetiformis is a rare type of generalized pustular psoriasis associated with pregnancy. Lesions in this type are itchy and may be foul smelling.

### Treatment of Psoriasis

Notably, there is no cure for psoriasis. However, various pharmacological and non-pharmacological options are available. Based on the severity of the condition, the treatment of psoriasis can be broadly divided into topical and systemic treatments. For the former, topical corticosteroids can be combined with vitamin D analogues. However, prolonged use of topical corticosteroids may lead to skin atrophy and they should not be used longer than recommended, usually not more than 8 weeks [43]. In some cases of psoriasis, phototherapy involving the use of ultraviolet light is applied [44]. For systemic therapy, drugs such as NSAIDs are mainly used in psoriatic arthritis whereas non-biologic disease-modifying drugs (DMARDs, e.g. cyclosporine, methotrexate) and biologics (e.g. interleukin inhibitors, TNF inhibitors, phosphodiesterase inhibitors) are used for both psoriasis and psoriatic arthritis [38, 39, 45].

## Overview of Mesenchymal Stem Cells

Mesenchymal stem cells (MSCs) are adult stem cells that were first described in 1996 by [46]. The bone marrow is a conventional and rich source of MSCs. However, MSCs can also be derived from other sources such as dental pulp, umbilical cord blood, adipose tissue etc [6]. Due to their multipotent potential, MSCs have been shown to differentiate into cells with mesodermal, endodermal, and neuroectodermal properties [8, 9]. When cultured in the lab and viewed under the microscope, these cells may take one of the three morphological patterns i.e. star-like shaped, elongated and spindled-shaped or cuboidal, flattened pattern [47]. Research has shown that MSCs derived from different sources may exhibit different biological properties e.g. the growth rate and secretory profile of proliferative cytokines were shown to differ in umbilical cord and skin-derived MSCs [48]. Although there is no standard protocol to isolate and expand MSCs in the lab, these cells are relatively easy to culture in general.

### Therapeutic Potential of MSCs in Inflammatory Diseases

MSCs’ therapeutic potential lies in the multipotent nature of MSCs, their ability to secrete trophic factors, as well as their immunomodulatory properties. There is increasing evidence that the role of MSCs in tissue repair and regeneration relies on their immunomodulatory properties and secretomes rather than the replacement of damaged cells. In inflammatory diseases, these properties are particularly useful in combating overwhelming inflammation.

### Secretory Profile of MSCs

MSCs are known to produce a vast array of secretory products including chemokines, cytokines, and growth factors. These soluble factors may exert an autocrine effect or act in a paracrine fashion. Some examples of these soluble factors include hepatocyte growth factor (HGF), fibroblast growth factor (FGF), interleukins (IL), insulin-like growth factor 1 (IGF-1), monocyte chemotactic protein-1 (MCP-1), vascular endothelial growth factor (VEGF), nerve growth factor (NGF) and many more [49, 50].

Recently, there has been a growing body of research on MSC-derived extracellular vesicles (MSC-Evs). Evs are membrane-bound nanoparticles containing diverse classes of substances such as lipids, proteins, nucleic acids and metabolites. Evs can be further classified into apoptotic bodies, exosomes, and microvesicles [51]. Some of the contents of MSC-Evs include growth factors, cytokines, mRNAs, and regulatory microRNAs (miRNAs). Therefore, some believe that MSCs are capable of exerting the effects of the parent cells via MSC-Evs without cell-cell contact, which renders MSCs a new potential cell-free therapeutic alternative [52]. Some advantages of MSC-Evs include their ability to cross biological barriers, higher safety profile, and lower immunogenicity. Research has shown that MSC-Evs exert anti-inflammatory effects in mice with experimental autoimmune encephalomyelitis through the regulation of pro- and anti-inflammatory cytokines [53] while MSC-MVs demonstrated a reduced and delayed inflammatory response in rats with rheumatoid arthritis [54].

### Immunomodulatory Properties of MSCs

The immunomodulatory properties of MSCs on cells of the innate and adaptive immune systems have allowed them to be one of the leading choices in the treatment of immune-mediated diseases. For example, MSCs sourced from different locations (placenta, bone marrow and umbilical cord) have been shown to exert immunosuppressive effects on T-cells in vitro. Co-culture experiments showed inhibition of CD4+ and CD8+ activated T-cell proliferation in via cell-cell contact [55]. Cell interactions and cross immunomodulation have been reported between MSCs and natural killer cells [56]. On the other hand, human amnion-derived MSCs delayed graft-versus-host disease (GVH) in mice by reduction in TNF-α and inhibition of T-cell activation and proliferation via the programmed cell death protein-1 (PD-1) receptor pathway [57].

It is worth mentioning that MSCs may play a role in the pathogenesis of some immune-mediated diseases. For example, MSCs in patients SLE have been reported to be defective in immunosuppression and immunomodulation. Research has shown that autologous MSCs fail to alleviate symptoms of SLE. On the contrary, MSCs transplanted from healthy donors may be beneficial and help reduce symptoms in patients with SLE [58].

Interestingly, MSCs’ reactions in inflammation can be bidirectional and hence they are not always immunosuppressive. In conditions where there is an under-activated immune system, MSCs can promote inflammation and when inflammation is overwhelming, MSCs can exert their anti-inflammatory effects. Kaundal et al. [59] demonstrated that immunomodulatory effects of MSCs could be polarised depending on toll-like receptor (TLR) priming. When MSCs were TLR-4 primed (*MSC1*), they expressed proinflammatory mediators predominantly whereas when TLR-3 primed (*MSC2*), they mainly expressed anti-inflammatory mediators. Co-cultures of peripheral blood mononuclear cells (PBMCs) with *MSC1* led to T-lymphocyte activation and with *MSC2*, led to T-lymphocyte suppression.

## Applications of MSCs in the Treatment of Psoriasis

Research has shown that MSCs are a potential therapeutic strategy in psoriasis, with increasing evidence reported in several in vitro, animal and clinical studies. This section will critically examine these studies and consolidate the key findings.

## Preclinical Studies

Zhang et al. [60] reported that imiquimod- (IMQ) or IL-23-induced psoriasis-like skin inflammation in mice was inhibited with the injection of human umbilical cord blood-derived mesenchymal stem cells (hUCB-MSCs). Some key findings of the study include decreased proinflammatory cytokine (e.g. TNF-α, IL-6, IL-17) and chemokine (CC17, CCL20, CCL27) expression in the skin. In vitro experiment, on the other hand, demonstrated that co-cultures MSCs inhibited CD4+T-cell activation and differentiation, an important aspect of psoriasis pathogenesis.

Superoxide dismutases are a group of enzymes with antioxidant effects. Research has shown that a decrease in superoxide dismutase is associated with increased oxidative stress in psoriatic lesions [61]. Subcutaneously injected superoxide dismutase (SOD)-transduced hUCB-MSCs, showed a reduction in disease severity, suppression of epidermal hyperproliferation and decreased acanthosis in IQM-induced psoriasis mouse model. The concentration of reactive oxygen species (ROS) was significantly lower in IMQ-treated mice using SOD-transduced MSCs or MSCs alone. There was also a decreased T-cell, neutrophil or dendritic cell accumulation in the back skin using SOD-transduced MSCs [62].

In vitro studies demonstrated suppression of T-cell proliferation, when CD4+ and CD8+T-cells were co-cultured with SOD-transduced MSCs. A greater level of suppression on proinflammatory cytokines and chemokines was observed using SOD-transduced MSCs when compared to MSCs alone. Inhibition of several signalling pathways [e.g. toll-like receptor-7 (TLR-7), nuclear factor-kappa B (NF-κB), p38 mitogen-activated kinase (MAK), and Janus kinase–signal transducer and activator of transcription (JAK-STAT), as well as adenosine receptor activation (ARA)] was implicated [63].

Human embryonic stem cells-derived (hESC)-MSCs suppressed T-cell reactivity against alloantigens when co-cultured with PBMCs. When administered subcutaneously, hESC-MSCs reduced dermatitis in IMQ-induced psoriasis in mice, with a reduction in splenomegaly. Histological improvement in psoriatic skin lesions was also evident, with a significant reduction in epidermal thickness. Several inflammatory cytokines (e.g. INF-α, IFN-γ, TNF-α, IL-17A, IL-23 and IL-27) were reduced in both the serum and skin of psoriatic mice [64].

In another study, infused human umbilical cord-derived (hUC)-MSCs demonstrated a reduction in psoriasis severity and development in IMQ-induced psoriasis mouse model. Suppression of immune cell infiltration to the skin and downregulation of proinflammatory cytokine expression (IL-1β, IL-6, IL-17 and IL-23) and markers of keratinocyte differentiation (S100A7, S100A8, and S100A9) were also observed, with upregulation of IL-10 (anti-inflammatory cytokine), neutrophil function suppression and downregulation of plasmacytoid dendritic cell type I interferon production (IFN-I) [65].

Zhang et al. [66] reported that topical application of hESC-MSC exosomes was shown to significantly reduce IL-7 (*p* = 0.05) and terminal complement activation complex C5b-9 (*p* = 0.00) in the mouse skin. In a human skin explant experiment, applied MSC labelled with fluorescence predominantly localised in the stratum corneum with little or no further penetrance to the stratum granulosum. It was proposed that the applied MSC exosomes might have suppressed complement activation in the stratum corneum layer of the skin and inhibited the release of IL-17 by neutrophils, as neutrophil infiltration into stratum corneum is a characteristic feature of psoriasis and these cells are a rich source of IL-17 in psoriasis.

In IMQ-induced psoriasis mouse model, infiltration of T-cells was reduced by intradermal injection of human adipose-derived (hAD)-MSCs, hUC-MSCs and human placenta-derived (hP)-MSCs in psoriatic skin lesions. Such reduction in T-cell infiltration, in turn, resulted in decreased IL-17 production by T helper (Th)-17 cells. Consequently, there was decreased keratinocyte proliferation in psoriatic skin lesions, which explains the reduction in epidermal thickness in the mice. These findings suggest that hAD-MSCs, hUC-MSCs and hP-MSCs exert inhibitory effects on proinflammatory Th-17 cells in psoriasis development [67].

In another study, hUCB-MSCs-treated mice showed normal skin with no erythema whereas untreated psoriatic mice showed erythema and scaling. These mice also showed uniform epidermal thickness with normal epidermal layers. The epidermis-dermis border was well-demarcated and the basement membrane was intact. There was absence of gaps between cells in the basal layer and resting basement membrane. On the other hand, untreated psoriatic mice showed epidermal hyperplasia, irregular and discontinuous basement membrane, as well as increased blood vessel count and immune cell infiltration [68].

Dental pulp stem cells (DPSCs) are undifferentiated MSCs derived from the neural crest. In IMQ-induced psoriasis mouse model, hepatocyte growth factor (HGF) overexpressed-DPSCs were reported to ameliorate erythema, scaling and thickening of psoriatic skin lesions, as well as reduce splenic mass in mice. Co-culture experiments with PBMCs showed downregulation of Th-1 and Th-17 cells and upregulation of regulatory T (Treg) cells. HGF-DPSCs also showed downregulation of cytokeratin (CK) 6, CK17, IFN-γ, IL-17A, IL-17F, IL-23, T-box transcription factor 21 (T-bet), retinoic acid-related orphan receptor-γt (RORγt), and upregulation of Foxp3 and IL-10 in psoriatic skin lesions, whereas downregulation of INF-γ, IL-17A and TNF-α in blood serums was also observed [69].

Ye et al. [70] investigated the therapeutic effects of human gingiva-derived MSCs (hGMSCs) in IMQ-induced psoriasis mouse model. Infusion of hGMSCs in mice improved skin inflammation by suppressing several pro-inflammatory cytokines related to Th-1 and Th-17, such as IL-1, IL-17A, IL-17F, IL-21 and IL-22, as well as TNF-α and IFN-γ. Results also showed that hGMSCs increased the percentage of spleen IL-17+CD3+ regulatory T (Treg) cells and decreased the percentage of spleen IL-17+CD3+T-cells (a type of Th-17 cells), suggesting that hGMSCs are exhibit therapeutic effects in psoriasis.

Exosomes derived from MSCs have been shown to be beneficial in the treatment of psoriasis. Zhang et al. [60] investigated the therapeutic effects of exosomes derived from human umbilical cord MSC-derived exosomes (hUC-MSCs-Exos) in IMQ-induced psoriasis mouse model and demonstrated significant suppression of epidermal proliferation and a reduction in Psoriasis Area and Severity Index (PASI) scores. The expression of pro-inflammatory cytokines and chemokines (e.g. IL-17, IL-23 and CCL20) were reduced while the phosphorylation of STAT3 was inhibited. Co-culture experiments demonstrated that hUC-MSCs-Exos suppressed the activation and maturation of dendritic cells and decreased the IL-23 expression levels. Collectively, the findings suggest that hUC-MSCs-Exos effectively improved skin inflammation in psoriasis through their immunomodulatory effects and by regulating the expression of IL-17 and IL-23.

Shi et al. [71] studied the effects of hAD-MSCs with and without vitamin E in IMQ-induced psoriasis mouse model. Infusion of hAD-MSCs was found to relieve the classical histological features of psoriasis in treated mice. Treatment with hAD-MSCs resulted in decreased pro-inflammatory cytokines, immune cell infiltration and splenic index. The therapeutic effects of hAD-MSCs were mediated via inhibition of reactive oxygen species (ROS) generation. A greater ROS inhibition was observed when mice were treated with the combination therapy of hAD-MSCs and vitamin E.

In IMQ-induced psoriasis mouse model, intravenous infusion of hUC-MSCs downregulated the expression of matrix metalloproteinase-13 (MMP13) in skin lesions. When co-cultured with THP-1 cells or phorbol myristate acetate (PMA)-stimulated THP-1 cells, hUC-MSCs showed anti-inflammatory effects as evidenced by reduced TNF-α levels in the co-culture supernatant. As TNF-α has been shown to upregulate MMP13 via the NF-κB pathway in keratinocytes, the study concluded that the therapeutic effects of hUC-MSCs were mediated through the NF-κB pathway [72].

**Fig. 1:**
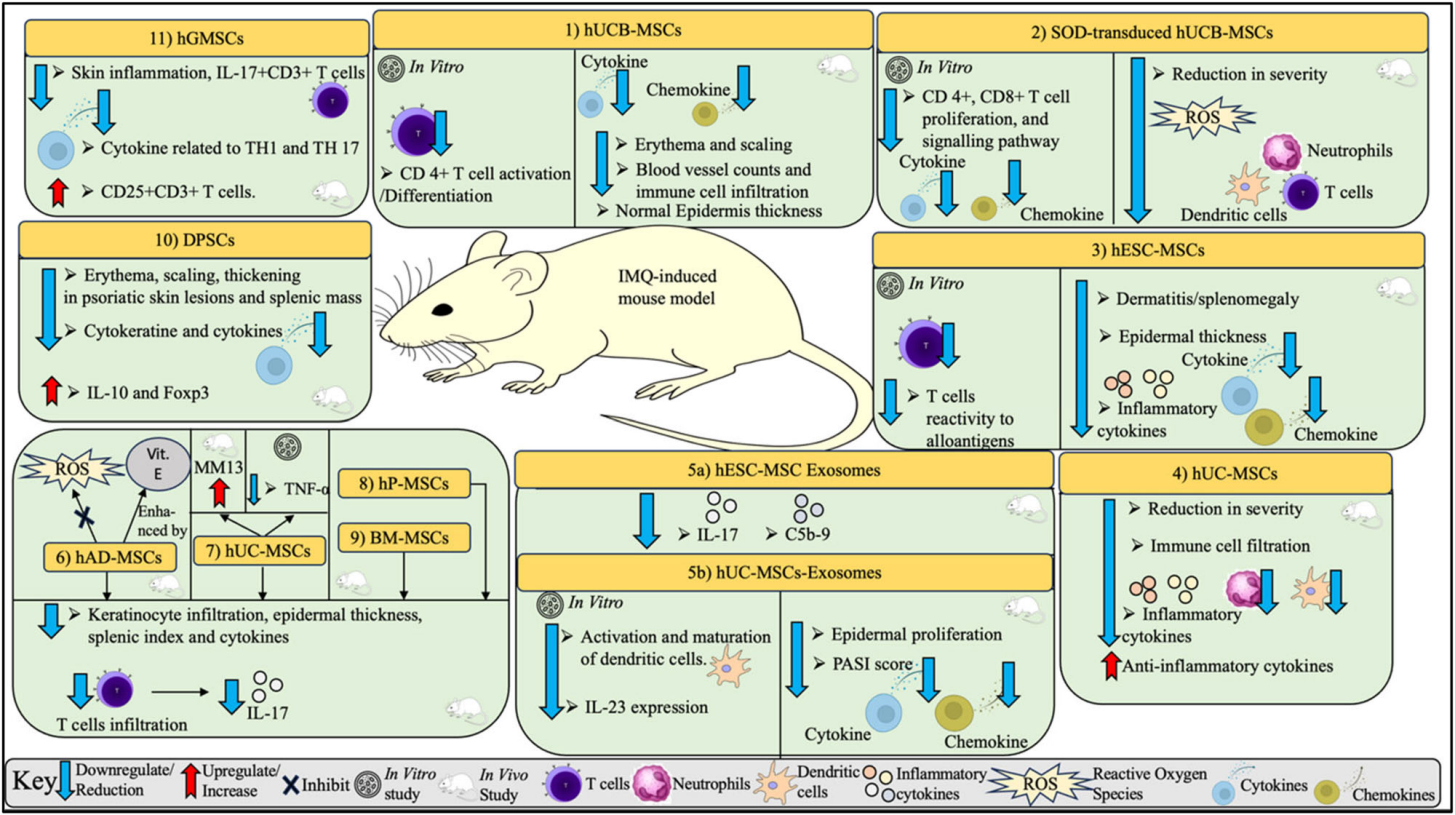
Summary of MSC-based Psoriasis Treatment in Selected In Vitro and In Vivo Studies

Cuesta-Gomez et al. [73] compared the therapeutic effects of BM-MSCs and AD-MSCs with and without cytokine pre-challenge (i.e. “licensed” and “unlicensed” MSCs) in an IMQ-induces psoriasis mouse model. Both licensed and unlicensed MSCs were reported to reduce the severity of psoriatic lesions. MSCs reduced T-cell infiltration and epidermal thickness, as well as promoted the production of immunomodulatory and pro-regenerative molecules, namely IL-17A and TGF-β. However, unlicensed MSCs were found to be more efficient in resolving skin inflammation. Table 3 and Fig. 1 provide a summary of the key results from preclinical studies investigating the therapeutic potential of MSCs in psoriasis.

**Table 3:** Summary of MSC-based Psoriasis Treatment in Selected Preclinical Studies

Study/ MSC type	Key findings	Reference
In vitro study and IMQ or IL-23-induced psoriasis mouse model/ hUCB-MSCs	In vitro • Co-cultured MSCs inhibited CD4+T-cell activation and differentiation In vivo • Inhibition of proinflammatory cytokine and chemokine expression by injected MSCs in skin of mice	[60]
In vitro study and IMQ-induced mouse model/SOD-transduced hUCB-MSCs	In vitro • Suppression of CD4+ and CD8+T-cell proliferation • Suppression of proinflammatory chemokines and cytokines • Inhibition of signaling pathways (TLR-7, NF-κB, p38 MAK, JAK-STAT, ARA) In vivo • Reduction in disease severity and acanthosis • Suppression of epidermal hyperinfiltration • Reduction in ROS • Reduction in T-cell, neutrophil or dendritic cell accumulation in the back skin	[62, 63]
In vitro study and IMQ-induced psoriasis mouse model/ hESC-MSCs	In vitro • Reduced T-cell reactivity to alloantigens in PBMC co-cultures In vivo • Subcutaneous injection of hESC-MSCs • Reduction of dermatitis and splenomegaly • Reduction in epidermal thickness • Reduction in INF-α, IFN-γ, TNF-α, IL-17A, IL-23 and IL-27 in skin lesions and serum	[64]
IMQ-induced psoriasis mouse model/ hUC-MSCs	• Reduction in psoriasis severity and development • Suppression of immune cell infiltration to skin • Downregulation of proinflammatory cytokines and markers of keratinocyte differentiation • Upregulation of anti-inflammatory cytokine • Neutrophil function suppression • Downregulation of IFN-I production	[65]
IMQ-induced psoriasis mouse model and human skin explant/ human embryonic stem cell-derived MSC exosomes	In vivo • Topical application of MSC exosomes showed significant reduction in IL-17 and C5b-9 in mouse skin Ex-vivo • Penetration of MSC exosomes limited to stratum corneum and little or no penetrance to underlying stratum granulosum	[60]
IMQ-induced psoriasis mouse model/ hAD-MSCs, hUC-MSCs an hP-MSCs	• Intradermal injection of hAD-MSCs, hUC-MSCs and hP-MSCs • Reduction in T-cell infiltration in psoriatic lesions leading to reduction of IL-17 production • Reduction in keratinocyte proliferation and dermal thickness in mice	[67]
IMQ-induced psoriasis mouse model/ hUCB-MSCs	• Subcutaneous injection of hUCB-MSCs • Reduction in erythema and scaling • Normal and uniform epidermis thickness (versus epidermal hyperplasia in untreated mice) • Intact basement membrane (versus irregular and discontinuous basement membrane in untreated mice) • Reduced blood vessel counts and immune cell infiltration	[68]
IMQ-induced psoriasis mouse model/ DPSCs	In vitro • Downregulated Th-1 and Th-17 and upregulated Treg in co-cultures with PBMCs In vivo • Reduction in erythema, scaling and thickening in psoriatic skin lesions • Reduction in splenic mass • Blood serum: downregulation of INF-γ, TNF-α, IL-17A • Skin lesions: downregulation of CK6, CK17, T-bet, IFN-γ, IL-17A, IL-17F, IL-23 and RORγt, • Skin lesions: upregulation of IL-10 and Foxp3	[69]
IMQ-induced psoriasis mouse model/ AD-MSCs	• Infusion of AD-MSCs relieved classical histological features of psoriasis in mice. • AD-MSCs reduced pro-inflammatory cytokines, immune cell infiltration and splenic index in treated mice. • Therapeutic effects of AD-MSCs were mediated through inhibition of ROS generation. • Effects of hAD-MSCs were enhanced when used together with vitamin E	[71]
IMQ-induced psoriasis mouse model/ hGMSCs	• Infusion of hGMSCs in mice ameliorated skin inflammation by: ➢ Reducing pro-inflammatory Th-1 and Th-17-related cytokines. ➢ Increasing the percentage of spleen CD25+CD3+T-cells. ➢ Decreasing the percentage of spleen IL-17+CD3+T-cells.	[70]
In vitro study and IMQ-induced psoriasis mouse model/ hUC-MSCs-Exos	In vitro • Suppression of activation and maturation of dendritic cells. • Decreased IL-23 expression levels. In vivo • Suppression of epidermal proliferation • Reduction in PASI scores • Reduced expression of proinflammatory cytokines and chemokines (IL-17, IL, 23 and CCL20)	[60]
In vitro study and IMQ-induced psoriasis mouse model/ hUM-MSCs	In vitro • hUC-MSCs co-cultured with THP-1 cells or PMA-stimulated THP-1 cells showed reduction in TNF-α levels in the co-culture supernatant. In vivo • IV infusion of hUC-MSCs downregulate MMP13 expression in skin lesions.	[72]
IMQ-induced psoriasis mouse model/ BM-MSCs and AD-MSCs	• Both licensed (challenged with cytokine) and unlicensed MSCs were shown to: ➢ reduce severity of lesions ➢ reduce T-cell infiltration and dermal thickness ➢ reduce epidermal thickness ➢ promote IL-17A and TGF-β production • Unlicensed MSCs were more efficient in resolving skin inflammation	[73]

*AD-MSCs* adipose-derived mesenchymal stem cells, *BM-MSCs* bone marrow-derived mesenchymal stem cells, *DPSCs* dental pulp stem cells, *ESC-MSCs* embryonic stem cell-derived mesenchymal stem cells, *GMSCs* gingiva derived mesenchymal stem cells, *IL* interleukin, *IMQ* imiquimod, *INF* interferon, *IV* intravenous, *MMP* matrix metalloproteinase-13, *ROS* reactive oxygen species, *TGF* tumour growth factor, *TNF* tumour necrosis factor, *UC-MSCs-Exo* umbilical cord mesenchymal stem cell-derived exosome, *UC-MSCs* umbilical cord-derived mesenchymal stem cells, *UCB-MSCs* umbilical cord blood-derived mesenchymal stem cells

## Clinical Studies

In 2016, Chen et al. [74] reported two cases in which treatment with umbilical cord-derived mesenchymal stem cells (UC-MSCs) resulted in complete remission of psoriasis vulgaris. The first case involved a 35-year-old man with psoriasis and B-cell lymphoma. The patient received chemotherapy, autologous haematopoietic stem cell transplantations (auto-HSCT) and one infusion of UC-MSCs to support engraftment of auto-HSCT. Both lymphoma and psoriasis went into remission with no relapse after five years of follow up. The second case was a 26-year-old woman given three weekly infusions of UC-MSCs followed by two infusions three months later. The patient remained psoriasis-free during the 4-year follow up period. The proposed effects of MSCs in these cases include (1) migration of MSCs to skin lesions, (2) immunomodulation, (3) autoimmunity limitation and (4) local paracrine effects of MSCs.

Infusions of autologous hAD-MSCs have been shown to improve psoriasis and psoriatic arthritis. A 58-year-old man received two infusions of hAD-MSCs showed a drop of Psoriasis Area and Severity Index (PASI) score from 21.6–8.9, with improvements in induration, erythema, onycholysis and psoriatic lesions. Reduction in PASI was sustained for up to 157 days. Further treatment with etanercept was given 10 months post MSC therapy, with remarkable improvements in scaling, psoriatic lesions and arthritis, and infliximab was given a month later, which resulted in minimal remaining erythematous plaques involving 10% of the body surface. A relapse of psoriasis and psoriatic arthritis was reported two years later when the patient contracted pulmonary tuberculosis [75].

In the same case report, a 28-year-old female with psoriasis vulgaris was given three infusions of hAD-MSCs. In the absence of her usual medications, the patient started with a PASI of 24 when receiving her first infusion, which dropped to 18.3 and 9.4 after the second and third infusions respectively. Her PASI was maintained at 9.6 for 45 days without methotrexate and she resumed methotrexate treatment 292 days post MSC therapy after a relapse [75].

Stromal vascular fraction (SVF) consists of a mixture of cells such as MSCs, endothelial precursor cells, fibroblasts, pericytes etc. obtained from adipose tissue. SVF is known to be rich in a wide range of growth factors and cytokines. Administration of SVF (obtained through a mini-lipoaspirate procedure under local anaesthesia) in a 43-year-old male patient with psoriasis resulted in improvements in psoriatic symptoms and quality of life over a 12 month-follow up. A remarkable drop in PASI from 50.4 to 0.3 was observed as early as one-month post IV SVF injection and the patient was drug free throughout the follow up period with no reported adverse effects [76].

The use of medium conditioned with hAD-MSCs has been shown to ameliorate psoriasis vulgaris. When the conditioned medium (CM) was applied to psoriatic erythematous plaques on the scalp of a 38-year-old male patient, there was significant reduction in silvery scales within two weeks. Complete disappearance of the lesions was observed within one month of the topical treatment, with a reduction of the Psoriasis Scalp Severity Index (PSSI) score from 28 to zero. Disease regression continued throughout the 6-month follow up period with no other medication. There were no adverse effects reported in the study and the effects of hAD-MSCs were believed to be due to cytokines, chemokines or growth factors present in the CM [77].

In another case report, infusion of allogenic gingival (G) MSCs demonstrated gradual clearance of psoriatic plaques in a 19-year-old male patient after two infusions a week apart. This was followed by three weekly infusions five weeks after the second infusion. The patient reported complete disappearance of the psoriatic lesions one week after the last infusion, with no relapse during the three-year follow up period [78].

Using one dose of intravenous and two doses of local transplantation of uncultured, minimally manipulated umbilical cord-derived (MM-UC)-MSCs, Ahn et al. [79] demonstrated a reduction in PASI score from 9.9–1.7 and a decrease in Dermatology Life Quality Index (DLQI) score from 27 to three, in a 47-year-old man with psoriasis 122 days after the first transplantation. There were remarkable improvements in symptoms such as erythema and itching. The disease did not recur during the five-month follow up period and the patient was medication-free during and after the MSC transplantation.

In a phase 1/2a study, Cheng et al. [80] infused human UC-MSCs in 17 patients and reported that the treatment was well-tolerated. Eight out of 17 patients (47.1%) experienced at least 40% improvement in their psoriasis symptoms, as measured by the PASI score while 17.1% (*n* = 3) of patients had minimal or no sign of disease, as measured by the PGA score. An increase in the levels of regulatory T-cells (Tregs) and CD4+ memory T-cells levels was observed, whereas the levels of Th-17, as well as serum IL-17 levels were reduced, suggesting the anti-inflammatory effects of MSCs in the treatment of psoriasis. On the other hand, patients who responded to MSC treatment showed a significantly lower level of Treg compared to those who did not respond to treatment, indicating the potential use of Treg levels as a biomarker to predict the efficacy of MSC treatment for psoriasis.

In a phase I clinical trial, Bajouri et al. [81] investigated the therapeutic effects of subcutaneously administered adipose-derived mesenchymal stromal cells (AD-MSCs) in five subjects and reported no major adverse effects. Psoriatic plaques were improved slightly to moderately after AD-MSC injection. Dermal mRNA expression of pro-inflammatory factors was reduced while Foxp3 transcription factor expression levels in the patients’ blood samples were increased, suggesting immunomodulatory effects of AD-MSCs. On follow-up six months post-injection, the patients reported no major adverse effects. On the other hand, a reduction in the PASI score, scaling of the plaques, erythema, and skin thickness was observed. Table 4 provides a summary of the key results from clinical studies investigating the therapeutic potential of MSCs in psoriasis.

**Table 4:** Summary of MSC-based Psoriasis Treatment in Selected Clinical Studies

Study/ MSC type	Key findings	Reference
Case report/ UC-MSCs	*Case 1* • Single dose of UM-MSCs in a patient with B-cell lymphoma and psoriasis after chemotherapy and autologous HSCT. • Complete remission for lymphoma and psoriasis for 5 years *Case 2* • Three weekly infusions of UM-MSCs followed by 2 infusions after 3 months • Complete remission of psoriasis for 4 years	[74]
Case report/ autologous hAD-MSCs	*Case 1* • Two infusions of hAD-MSCs • Reduction in PSAI from 21.6–9.9 (for 157 days) • Improvements in induration, erythema, psoriatic lesions and onycholysis • Further improvements in psoriasis and psoriatic arthritis with additional treatment (etanercept and infliximab) *Case 2* • Three infusions of hAD-MSCs • Drop of PSAI from 24 (1st infusion) to 18.4 (2nd infusion) and 9.4 (3rd infusion) • 292 days without methotrexate post MSC therapy and resumed methotrexate following a relapse.	[75]
Case report/ SVF	• Improvements in psoriatic symptoms and quality of life • PSAI decreased from 50.4 to 0.3 one-month post treatment • Drug free during 12-month follow up	[76]
Clinical study (Case report)/ hAD-MSCs	• Application of hAD-MSCs CM on scalp • Reduction of silvery scales within 2 weeks • Disappearance of erythematous plaques within 1 month • Reduction of PSSI score from 28 to 0 • No reported adverse side effects • Effects may be due to cytokines, chemokines or growth factors in CM	[77]
Case report/Allogenic G-MSCs	• Two weekly infusions of G-MSCs followed by three weekly infusions after 5 weeks • Complete clearance of psoriatic plaques one week post treatment and no relapse during 3-year follow up	[78]
Case report/ MM-UC-MSCs	• One dose of IV MSCs and two doses of local MSC transplantation • Reduction of PASI from 9.9 to 1.7 • Reduction in DLQI from 27 to 3 • Treatment-free remission post MSC transplantation	[79]
Phase 1/2a clinical trial/ hUC-MSCs	• Treatment was well tolerated in 17 patients. • Improvement in PASI and PGA scores • Anti-inflammatory effects with increased levels regulatory T-cells (Tregs) and CD4+ memory T-cells and decreased levels of Th-17 and IL-17. • Potential of using Treg level as a biomarker to predict treatment efficacy	[80]
Phase 1 clinical trial/ AD-MSCs	• Treatment was well tolerated in 5 patients. • Subcutaneous injection of AD-MSCs resulted in slight to moderate improvement of psoriatic plaques. • Reduction in dermal mRNA expression of pro-inflammatory factors • Increase in blood Foxp3 transcription expression levels • Reduction in PASI score, a reduction in PASI score, scaling of the plaques, erythema and skin thickness was observed on 6-month follow up.	[81]

*AD-MSCs* adipose-derived mesenchymal stem cells, *CM* conditioned medium, *GMSCs* gingiva-derived mesenchymal stem cells, *MM-UC-MSCs* minimally manipulated umbilical cord-derived mesenchymal stem cells, *PASI* psoriasis area and severity index, *PGA* physician’s global assessment, *SVF* stromal vascular fraction, *UC-MSCs* umbilical cord-derived mesenchymal stem cells

## Conclusions

Psoriasis is a common multi-factorial inflammatory skin disease without a curative treatment. Conventional treatment of psoriasis revolves around pharmacotherapy and phototherapy. Despite the availability of many drugs, drug-free remission is sometimes hard to achieve in severe cases and the quality of life is much affected. In recent years, numerous preclinical and clinical studies have shown that MSCs are potential candidates for cell-based therapy in psoriasis. Various types of MSCs (e.g. DPSCs, G-MSCs, hAD-MSCs, hESC-MSCs, hUC-MSCs, hUCB-MSCs, hP-MSCs etc) have been used in these studies. Other than using the MSCs directly, conditioned medium, stromal vascular fraction and MSC-derived exosomes have also been applied.

The MSCs, their conditioned medium or cell products have been administrated via different routes of delivery such as intravenous injection, intradermal injection, subcutaneous injection, and topical application on skin lesions. Research has shown that MSCs from various sources are capable of improving psoriasis in experimental animals and humans with little or no adverse effects. Such improvements include reduction of symptoms that are clinically and histologically evident and improved quality of life. A reduction in immune cell infiltration in psoriatic skin lesions and pro-inflammatory mediators and enhancement of anti-inflammatory mediators are frequently observed. Some patients treated with MSCs remain symptom- and drug- free for months or even years in these studies. Such findings suggest that MSCs have high therapeutic values in the treatment of psoriasis. However, many of the clinical studies involved a small number of patients and were mostly reports of sporadic cases. Future research should involve larger clinical trials and focus on the underlying mechanisms of how MSCs alleviate psoriasis.

## Data Availability

No datasets were generated or analysed during the current study.

## Abbreviation

AD-MSCs: Adipose-derived mesenchymal stem cells
BCG: Bacillus Calmette–Guerin
BM-MSCs: Bone marrow-derived mesenchymal stem cells
CCL20: C-C-motif ligand 20
CM: Conditioned medium
DM: Diabetes mellitus
DMARDs: Disease-modifying drugs
DPSCs: Dental pulp stem cells
ESC-MSCs: Embryonic stem cells-derived mesenchymal stem cells
Exo: Exosomes
EV: Extracellular vesicles
FGF: Fibroblast growth factor
GMSCs: Gingiva derived mesenchymal stem cells
GWAS: Genome-wide association study
HGH: Hepatocyte growth factor
IGF-1: Insulin-like growth factor 1
IL: Interleukin
IMQ: Imiquimod
INF: Interferon
IV: Intravenous
MAK: Mitogen-activated kinase
OMIM: Online Mendelian Inheritance in Man
PASI: Psoriasis area and severity index
PSORS: Psoriasis susceptible
MM-UC-MSCs: minimally manipulated umbilical cord-derived mesenchymal stem cells
MMP: Matrix metalloproteinase-13
MS: Multiple sclerosis
NGF: Nerve growth factor
PGA: Physician’s global assessment
RA: Rheumatoid arthritis
ROS: Reactive oxygen species
SLE: Systemic lupus erythematosus
TGF: Tumour growth factor
TLR: Toll-like receptor
TNF: Tumour necrosis factor
UC-MSCs-Exo: Umbilical cord mesenchymal stem cell-derived exosome
UC-MSCs: Umbilical cord-derived mesenchymal stem cells
UCB-MSCs: Umbilical cord blood-derived mesenchymal stem cells
VEGF: Vascular endothelial growth factor
WHO: World Health Organisation
